# Supplementary material for: Patterns of Intron Gain and Loss in Fungi
Source: PLoS Biol. 2004 Nov 30;2(12):e422. doi: 10.1371/journal.pbio.0020422 (PMC532390; doi:10.1371/journal.pbio.0020422)
Supplement: Table S1 — Also available at http://genes.mit.edu/NielsenEtAl/. (4.3 MB ZIP). [file pbio.0020422.st001.zip › NielsenEtAl/html/332.html]

AN1370.1.NCU07460.1.MG09882.1.FG00518.1


```
 CLUSTAL W (1.82) Multiple Sequence Alignments - Introns Inserted


Sequence 1: MG09882.1	301 aa
Sequence 2: FG00518.1	343 aa
Sequence 3: NCU07460.1	298 aa
Sequence 4: AN1370.1	290 aa
Alignment Length: 357 aa
Number Identitical Residues: 97 aa
Alignment Score (without introns) 6422


MG09882.1 	-----~-------------------------------------~----MASKLPPLPNSP
NCU07460.1	-----~----------------------------------MSW0SSANLSSKLPPLPASP
FG00518.1 	MSGTC2NARFRGGSGKASSGPQKSPTGSTASQPPPELPAQGNQ~SAASLAPKVPPLPSSP
AN1370.1  	-----~-------------------------------------~-MNNGSPGQSPAPQSP
          	                                               . :.  .* * **

MG09882.1 	SLAQTLGMDENSGIMATGEDILN--SYHLPRPLPLWLNPGYARHIVKGNFMTLSARPKTV
NCU07460.1	SLSQSIGM------MGTGEEALN--AYHLPRPLPLWLNPTAAKHIVKGNFMTLSARPKTV
FG00518.1 	SFSQTIGMDESSGVMS-GDDLIS--SYHLPRPLPLWLNAQYAKHIVKGNFMTLSARPKTV
AN1370.1  	HGHVPNAQS-----FASNHSAIETTAVYDPDAPKYFFQEKYAPLNVKGNFLTLCACPKNV
          	    . . .     :.:... :.::: : * .   :::   *   *****:**.* **.*

MG09882.1 	EQGEWIAHQV1VEHYRNLWNFVRVVHEK-EEDGTTICNSTTCPRTSAGA2N----HSFTW
NCU07460.1	EQGEWIAHQV1VEHYRNLWNFVRVVYEK-EEDGKSICNSITCPRMSAGT2NGHRNHSYTW
FG00518.1 	EQGEWIAHQV~IEHYRNLWNFVRVLHEK-EDDGTSICNSTSCPRMSAGT2N----HSFTW
AN1370.1  	ELGEWLAHQI1VEQNRLLQAMLKVIQEVNSHTGLPICNETSCPTMSAGR2L-----TYTW
          	* ***:***: :*: * *  :::*: * ... * .***. :**  ***        ::**

MG09882.1 	LNSRREPVELPAHEYMTLMQRWISGKIDDTNIFPTE-ASGVS--FAHNPQYCPPALSQLA
NCU07460.1	LNSRFEPIELPAYEYITLMQRWISGKIDDTKMFPTE-ASGVS--FAHNPQITTTAL--LT
FG00518.1 	LNRNREPVELPAHEYMTLMQRWISGKVDDTNIFPTD-PSGVS--YAHNPSITTTPLSQLS
AN1370.1  	LVDS-QAAKISAPKFINRVEKWIVSKIHDPVMFPTEKVTGVPDTFALSEATGASAADAAA
          	*    :. ::.* :::. :::** .*:.*. :***:. :**..::* .    ... .  :

MG09882.1 	GPGGEGGDWVGKRSGFPENFVEVCKTIFRQMFRVYAHLYWAHFVEPFYHLNLEKQLNSCF
NCU07460.1	GPD----DWIGKRSGFPKEFFNICQTIFLQMFRVYAHLYWAHFTEPFYHLNLEKQLNSCF
FG00518.1 	SPGEP--EYIGKRSGFPDKFVDICQMIFRQMFRVYAHLYWAHFSEPFYHLNLEKQLNSCF
AN1370.1  	NAGGE--DWIGKSSGFPRTFYKDCQGIMKQMFRCYAHLYHAHWLNPFWHINKHDILNMCF
          	...    :::** ****  * . *: *: **** ***** **: :**:*:* .. ** **

MG09882.1 	SHFVLTATALDMLKPHELEPMQPLIDLWAANGTFPQGSRAYEYANLRAGEKLMQLGGMST
NCU07460.1	SHFVLTATALDMLKPAELEPMQPLIDLWAANGTFPPESKAYEYANLKVGTRLMQMAGLA-
FG00518.1 	SHFVLTATALDMLKPAELEPMQPLIDLWAANGTFPPESKAYEYANIRAGERLLQLSTAPQ
AN1370.1  	VHFVTVAKYYKLVSDKEMEPMQPLIDLFIKQQRIPPEA-------LNGGHWGQQSSS---
          	 *** .*.  .::.  *:*********:  :  :*  :       :. *    * .    

MG09882.1 	S
NCU07460.1	-
FG00518.1 	-
AN1370.1  	-
          	
```
